# Capacity building for dementia care in community care services: a mixed methods approach

**DOI:** 10.1186/s12877-020-01517-8

**Published:** 2020-03-30

**Authors:** Helen Y. L. Chan, Florence K. Y. Ho, Kenny C. M. Chui, Eunice Y. S. Hui, Bel Wong, Yuen-yu Chong, Alison Bowes, Timothy C. Y. Kwok

**Affiliations:** 1 The Nethersole School of Nursing, Faculty of Medicine, The Chinese University of Hong Kong, Hong Kong SAR, China; 2 Jockey Club Centre for Positive Ageing, 27 A Kung Kok Street, Shatin, New Territories, Hong Kong SAR, China; 3 Dementia Services Development Centre, University of Stirling, Stirling, UK

**Keywords:** Dementia, Evaluation, Staff training, Capacity building, Reflective learning, Community care, Staff competence

## Abstract

**Background:**

The prevalence of dementia is surging that results in huge service demand in the community care services. Dementia care competence of staff working in these settings is fundamental of the care quality. This project aims to examine the effects of staff training on their competence for the anticipated challenges in dementia care and explore how the training influence their care practices.

**Methods:**

This study adopted a mixed methods triangulation design, including a prospective multi-center study with pre-test post-test evaluations and a narrative analysis of the participants’ reflective essays. Seventeen experienced health and social care professionals were trained as trainers at the Dementia Services Development Centre of the University of Stirling, UK. The trainers provided local facilitator training to staff members by using training materials that were culturally adapted to the local context. The facilitators were required to deliver 12 two-hour in-service training sessions for 6 months to their colleagues in a small group format in their respective workplace. Eventually a total of 1347 staff members from community care centers, day care centers, outreach teams and care homes of 70 non-government organizations in Hong Kong participated in the study between April 2017 and December 2018. Validated instruments were used to measure knowledge, attitude, sense of competence in dementia care and job satisfaction at the baseline and at 12-month follow-up. All participants were required to write a reflective essay to describe their experiences in dementia care by the end of the training.

**Results:**

A total of 1264 participants, including 195 facilitators and 1069 learners, completed all assessment were included for analysis. Significant improvements were observed in all outcomes at the 12-month follow-up assessment (*P*s ≤ .001). The magnitude of improvements in attitudes was the largest. The findings also showed that the effects of the training program significantly varied across different groups of learners in terms of age, occupation, work and training experience.

**Conclusions:**

This community-wide large-scale project provided evidence that the train-the-trainer model and reflective learning are effective means to facilitate situated learning that promote awareness and understanding of dementia, and consequently enhance sustainability of changes in care practices.

## Background

Dementia care training is a pressing global issue [1]. Compromised cognitive functioning deprives people with dementia the ability to express their thoughts or apprehend the information surrounding them, thus leading to various challenging behaviors. Studies show that health care providers perceive themselves as lacking confidence or skills in dementia care [2, 3]. Their inappropriate attitudes toward dementia negatively impact care quality, job satisfaction, relationships with clients and their family members [2, 4]. Hence, training is needed to enhance the knowledge, attitudes, skills, and confidence of staff in meeting the care needs of people with dementia [5–8].

Nearly 50 million people worldwide currently suffer from dementia. The incidence rate of newly diagnosed is expected to surge at about 10 million new cases per year [9]. With the rising service demand, the care for people with dementia is expanding beyond mental health specialist services or long-term care settings to general hospital and community care settings. Moreover, patients’ complex care needs require diverse support from staff of different levels, disciplines and expertise [10, 11]. However, existing evidence of dementia care training is predominant only in health professionals, nursing homes and Western countries and is limited by small sample size [7, 8, 12].

The current project aims to examine the effects of the Best Practice in Dementia Care Learning Program [13], a structured dementia training program in community care services in Hong Kong. Although this program is widely adopted in various care settings in European countries, only one paper report its effects [13] The findings suggested that participants gained additional knowledge about dementia and that the care practices improved after the training, but the outcomes were only measured by using a self-developed questionnaire among 100 participants.

The current project is built on the previous work to specifically [1] examine the effects of the program on staff knowledge, attitude, sense of competence related to dementia care and job satisfaction using validated instruments and [2] explore how the dementia care practices has been influenced by the training. Previous studies concluded that knowledge may not necessary translate into practices [7, 14]. Hence, the project evaluation included several staff outcomes. In this project, two sets of training kits were translated to Chinese and tailored according to the local sociocultural context in residential and community care services. To ensure holistic care, the program curriculum comprised the following domains: (i) dementia and persons with dementia; (ii) person-centered care and building meaningful relationship; (iii) communication and behaviors; (iv) support for people with dementia, family, and carers; (v) health and wellbeing; and (vi) legal aspects and issues related to dementia. The program adopted the train-the-trainer model. Seventeen local health and social care experts in aged care services received training at the Dementia Services Development Centre of the University of Stirling in Scotland. The trained personnel then delivered a series of three-day facilitator training workshops to experienced staff members in the local care settings. These staff members will serve as facilitators for the situated training for their colleagues. Each facilitator provided 12 two-hour training sessions for a group of around six staff members (i.e. learners) in their workplace for 6 months. This training program emphasized skilled facilitation and situated reflective learning within the workplace [13]. Therefore, the training sessions encompassed various interactive activities, such as case sharing, group discussion, and reflective exercises to encourage active learning. After completing 12 training sessions, all participants were required to write a reflective essay describing how they managed the challenging situations related to dementia. Reflective writing, which highlights the critical consideration of one’s experience, is an effective learning strategy in medical education because the deliberation process fosters the integration of new learning and existing knowledge [15].

## Methods

### Design and setting

This project adopted a mixed methods triangulation design in which quantitative and qualitative data were collected concurrently but separately [16]. The data were collected through a prospective multicenter study and a narrative analysis of participants’ written accounts (i.e., reflective essays). Such a design enabled researchers to further understand the phenomena by integrating the two sets of results for interpretation [16]. Given the community-wide quality improvement initiative, randomization was impractical. Hence, pre- and post-test designs were used for the program evaluation. Seventy non-government organizations, including community care centers, day care centers, residential care homes for elderly and outreach teams, participated in the study. All staff members working in these facilities were eligible to participate in the study. Eventually, 1347 staff members participated in the study. After completing the facilitator training, 218 facilitators provided dementia care training to 1129 staff members in their respective workplaces. Fifty written accounts were randomly sampled from different care settings on the basis of the proportion of participants to maximize the sample variation. The qualitative findings were used to confirm quantitative results for drawing a substantiated conclusion about the effects of the program. The value of qualitative study in evaluating dementia training is emphasized as a means of understanding how staff perceive learning gains that are difficult to measure [8]. Ethical approval for the study was obtained from the University Survey and Behavioural Research Ethics Committee.

### Data collection

A self-administered questionnaire was used to assess learners’ knowledge, attitudes, self-perceived competence in dementia, and job satisfaction before the training and at the 12-month post-enrolment. Data collection was conducted between April 2017 and December 2018. The Dementia Knowledge Assessment Scale (DKAS) was used to assess the dementia knowledge on four aspects, namely, causes and characteristics (7 items), communication and behavior (6 items), care considerations (6 items), and risk and health promotion (6 items) [17]. Participants were asked to judge the accuracy of the 25 statements by using five response options (false, probably false, probably true, true, or don’t know). The negatively phrased statements were recoded, and high scores signify greater knowledge in dementia care. The Dementia Attitudes Scale (DAS) was used to assess the affective, behavioral, and cognitive components of attitudes toward dementia [18]. DAS covered two domains, namely, dementia knowledge (10 items) and social comfort (10 items). Each item was rated on a seven-point Likert scale (1 = totally disagree to 7 = totally agree). The Satisfaction with Nursing Care and Work Assessment Scale (SNCW) was used to assess job satisfaction [19]. Participants were asked to indicate their level of agreement with 35 items related to cooperation, development, quality of care, workload, and knowledge of patients by using a five-point Likert scale. The scores of negatively phrased statements in DAS and SNCW were reverse coded; a higher score on the two measures suggest a more positive attitude toward dementia and a greater level of job satisfaction, respectively. The Sense of Competence in Dementia Care Staff (SCIDS) was used to measure the self-perceived competence in working with people with dementia [20]. Seventeen items were categorized into four domains, namely, professionalism (5 items), building relationships (4 items), care challenge (4 items) and sustaining personhood (4 items). Each item was rated on a four-point Likert scale (from 1 = not at all to 4 = very much), and higher scores indicate a higher sense of competence in dementia care. To reduce response burden, the questionnaire for facilitators only included the part on attitudes toward dementia and job satisfaction.

### Statistical analysis

The normality of continuous variables was assessed through skewness statistics and normal probability plots. The missing value of each variable was less than 10%. Little’s missing completely at random (MCAR) test revealed that the data were MCAR (χ^2^ = 63.61, *df* = 234), suggesting that no obvious pattern in the missing data. Therefore, multiple imputations using a fully conditional specification with at least five imputations were performed to replace the missing values [21]. In addition, descriptive statistics was used to summarize the data. Paired sample *t*-tests were used to assess the changes in outcome measures between the baseline and 12-month follow-up assessment, followed by examining the within-group effect sizes in terms of Cohen’s *d* using the formula used by G*Power. Univariate analyses, including independent sample *t*-tests and one-way analysis of variance (ANOVA), were performed to examine if the within-group difference in each outcome differed amongst the subgroups of learners. All statistical analyses were performed using IBM SPSS 25 (IBM Corp., Armonk, NY) and two-sided tests with the level of significance set at 0.05.

### Narrative analysis

In this study, 50 written accounts, including 20 pieces from care homes and 10 pieces from the other setting (community centers, day care centers and outreach teams), were sampled to explore how their care practices for people with dementia have been influenced after the training from their perspectives [22]. The authors independently read the written accounts to understand the participants’ experiences. Texts about similar experiences from different written accounts were extracted and condensed into meaning units for categorization. These qualitative findings were complementary for interpreting the changes in outcome measures over time [23].

## Results

### Participants’ characteristics

A total of 1264 participants, including 195 facilitators and 1069 learners, completed the baseline and 12-month follow-up assessments, resulting in a response rate of 93.8%. Table 1 presents the characteristics of the participants from a range of care settings, types of occupations and experiences in dementia care. The participants had an average of approximately 7 years of experience in aged care services. The majority (82.5%) of the participants were involved in direct services for people with dementia in their daily work. Many clerical staff (52.9%) and supporting staff (23.8%) considered that their duties were not directly related to dementia care. More than two-thirds of the participants received dementia care training in various formats, such as talks, seminars, or workshops before joining the program. Of which, most of the managerial staff (81.0%), professional staff (68.8%) and care assistants (69.5%) had received prior training whereas less than half of supporting staff (45.4%) and clerical staff (32.8%) had such preparation.

**Table 1:** Participants’ characteristics (*N* = 1264)

	Number	Valid %
Gender		
Male	200	15.8
Female	1039	82.2
Age (years)		
≤ 24	71	5.6
24–34	353	27.9
35–44	250	19.8
45–54	397	31.4
55–64	161	12.7
≥ 65	14	1.1
Types of care settings		
Community centers	255	20.2
Day care centers	275	21.8
Residential care homes	486	38.4
Others	246	19.5
Types of staff		
Care assistants	613	48.5
Supporting staffs	23	1.8
Clerical staffs	75	5.9
Professional staffs	486	38.4
Management staffs	29	2.3
Types of professional staffs (*n* = 486)		
Social work	202	15.9
Nursing	194	15.3
Allied health	38	3.0
Others	68	13.4
Year(s) of experience working in aged care service, *M* (*SD*)	Facilitators: 6.96 (6.25)	
	Learners: 7.27 (6.42)	
Provision of direct services to people with dementia in daily job duties		
Yes	1043	82.5
No	187	14.8
Ever received dementia training before this study		
Yes, more than once	435	34.4
Yes, once only	388	30.7
No	381	30.1

Footnotes: *N* total number, *n* number, *M* mean, *SD* standard deviation

### Dementia-related knowledge

The mean total DKAS and the four subscale scores of the learners significantly improved after training (*P*s ≤ .001) (Table 2). An increase in the total DKAS score was observed in 71.9% of the learners. The reflective essays revealed that certain participants knew little about dementia before receiving training. They failed to recognize the challenging behaviors exhibited by the clients were related to dementia and often found them uncooperative. For example, scolding others or suspecting their things had been stolen. The participants used to argue with the clients in an attempt to correct them but they remained defensive. One participant who worked in a care home shared that,

“*There was once when we were playing building blocks, an old lady put one piece into her mouth. We immediately tried to open her mouth to get it back when we noticed that. She was resistant and ran back to her room. This lady had the experience of picking the food from other resident’s dishes. At that time, I tapped on her shoulder to remind her wrongdoing, but she vituperated. Her reaction was totally intolerable.*”

**Table 2:** Knowledge, Attitudes, Sense of Competence and Job Satisfaction toward Dementia Care at baseline and 12-month follow up (*N* = 1264)

Measures	Possible range	Pretest, *M* (*SD*)	Posttest, *M* (*SD*)	Change in score, *M* (*SD*)	*t statistics*	Within-group ES, *d*
DKAS - Total score^b^	0–50	27.7 (8.6)	32.7 (7.6)	+ 5.0 (8.0)	20.4***	0.619
DKAS – Causes and characteristics^b^	0–14	7.7 (3.0)	9.0 (2.7)	+ 1.3 (3.0)	14.1***	0.438
DKAS – Communication and behavior^b^	0–12	5.7 (2.8)	7.2 (2.8)	+ 1.5 (3.0)	16.0***	0.495
DKAS – Care considerations^b^	0–12	8.3 (2.9)	9.2 (2.6)	+ 0.8 (3.1)	8.5***	0.288
DKAS – Risk factors and health promotion^b^	0–12	6.8 (2.7)	7.3 (2.6)	+ 0.6 (3.2)	5.9***	0.157
DAS – Total score^a^	20–140	108.2 (12.1)	117.3 (10.6)	+ 8.9 (9.2)	13.4***	0.983
DAS – Total score^b^		102.2 (12.0)	112.0 (11.2)	+ 9.7 (11.9)	26.6***	0.819
DAS – Dementia knowledge^a^	10–70	57.3 (5.8)	60.9 (4.9)	+ 3.6 (4.7)	10.7***	0.782
DAS – Dementia knowledge^b^		48.4 (7.9)	54.3 (7.0)	+ 6.0 (7.5)	25.9***	0.785
DAS – Social comfort^a^	10–70	50.7 (8.4)	56.3 (6.9)	+ 5.6 (6.6)	11.8***	0.871
DAS – Social comfort^b^		53.9 (6.1)	57.7 (5.5)	+ 3.7 (6.4)	19.2***	0.599
SCIDS – Total score^b^	17–68	43.2 (8.9)	48.3 (7.6)	+ 5.1 (8.2)	21.4***	0.619
SCIDS – Professionalism^b^	5–20	13.9 (3.2)	15.2 (2.8)	+ 1.3 (3.0)	13.6***	0.425
SCIDS – Building relationship^b^	4–12	9.3 (2.2)	10.6 (2.0)	+ 1.3 (2.4)	19.2***	0.545
SCIDS – Care challenges^b^	4–12	9.3 (2.4)	10.6 (2.1)	+ 1.3 (2.4)	17.9***	0.555
SCIDS – Sustaining personhood^b^	4–12	10.7 (2.4)	11.9 (2.0)	+ 1.2 (2.4)	17.4***	0.501
SNCW– Total score^a^	32–160	117.4 (13.3)	121.5 (14.0)	+ 4.1 (12.1)	4.8***	0.337
SNCW– Total score^b^		119.7 (13.7)	125.3 (14.6)	+ 5.6 (12.2)	14.6***	0.458

Footnotes: ^a^Number of facilitators = 195; ^b^Number of learners = 1069; *DKAS* The Dementia Knowledge Assessment Scale, *DAS* Dementia Attitudes Scale, *SCIDS* The Sense of Competence in Dementia Care, *SNCW* Satisfaction with Nursing Care and Work Assessment Scale, *d* Cohen’s d, *ES* effect size, *N* total number, *M* mean, *SD* standard deviation; *** denotes *p* < .001

Upon receiving the training, the participants were interested in exploring the reasons behind the behaviors or emotions exhibited by their clients.

### Attitudes toward dementia

The mean total DAS and the two subscale scores amongst facilitators and learners significantly improved (*P*s ≤ .001) (Table 2). An increase in the total DAS scores was noted in 80.5% of the participants. In their reflective essays, the participants shared that they started to appreciate the abilities of people with dementia after their training rather than merely focusing on their weaknesses and recognized the time needed for trust building. For example, some participants often kept their clients in armchairs or held their arms whenever they walked because they were worried that their clients may fall due to lower limbs weakness or visual impairment. However, their actions were not appreciated by these clients and in turn triggered unsafe or aggressive reactions, such as fleeing or pushing others. A participant shared that,

“*Mr Wong had left the center for several times himself and could not recognize the route back over the past year. Fortunately, every time he was brought back by some neighbors. We sometimes mocked him for his absent-mindedness and then he became short tempered. Once he asked me if he was being put under surveillance due to his misbehaviors.”*

The participants realized that people with dementia also have psychosocial needs. A participant highlighted in the essay the importance of being empathetic and paying attention to their psychosocial needs.

*She recalled an incident that an old lady had wet her pants when she was walking away during a group activity. Her colleague noticed and shouted, “Ms. Lee, you have wet your pant. Please don’t move around and I will go to get another pair of trousers for you to change.” When she assisted Ms Lee back to the seat, she further said, “You have already wet your pants once this morning.” At that moment, everyone turned silent and looked at Ms. Lee who denied that she had done so. She replied irritably, “Don’t call my daughter. I haven’t wet my pants. I was just walking to the toilet...Get off from me! I don’t need your help!” Ms Lee shook her colleague off and walked away. They helped her to change the trousers eventually. However, she remained unhappy throughout the day, even though she cannot recall what evoked the negative emotion.*

After the training, the participant reflected that people with dementia may also have the feeling of embarrassment. Hence, she discussed strategies for preserving their dignity in the essay. Another participant also shared her experience wherein she was once rejected by an old man for accompanying him to the toilet, as he was concerned about the gender difference.

### Sense of competence in dementia care

The mean total SCIDS and the four subscale scores of the learners significantly improved after training (*P*s ≤ .001) (Table 2). An increase in the total SCIDS scores was observed among 73.4% of the learners. Many participants shared in the reflective essays that they were greatly confident in interacting with people with dementia. Many examples on how the participants attempted to design different kinds of activities in relation to previous work experiences or the specific hobbies of the people with dementia were gathered. One participant shared her experience in managing the challenging behaviors of her client.

*Mr. Chan was restless in the community center and kept on shouting that he wanted to leave and went to the restaurant that he owned. Initially, the staff members attempted to orient him the purpose of attending the center, but he became unhappy as he perceived himself useless. His family members were frustrated because his behaviors persisted at home and can hardly be settled.*

Following the training, the participant attempted to learn more about his life story and then design activities that matched his experience. She stated that,

*Instead of calling him “Uncle Chan,” she called him “Boss Chan.” I tried to invite him to write menu. He was very delighted to do so and became very concentrated in the planning and writing process.*

The participant underscored knowing the person is an effective way to understand the reasons behind the challenging behaviors exhibited by people with dementia. Likewise, another participant also shared her positive experience on how to engage an old man who was used to yelling out profanities in the center.

“*We have tried to think about the solution for months. We invited him to read aloud the newspapers at least this can prevent him from using swear words. Recently, we tried to change the text to Tang poems (a kind of Chinese classical literature). We can’t imagine that he can even tell us the meaning of the ancient words, apart from reading it out. He became courteous and patience. This is the first time I found that we can communicate with him.*”

Other participants also noted that they engaged their clients according to their interests.

### Job satisfaction

The mean SNCW score of the facilitators and learners significantly improved after the training (*p* ≤ .001) (Table 2). Around two-thirds of the participants (66.5%) reported a higher level of job satisfaction than the baseline after training. In the reflective essays, some of the learners valued an open atmosphere for active sharing in the training sessions, and an improvement in collegial relationship and team collaboration was observed. Here is a quote from their essays,

*“The group sessions held regularly in our workplace provided us an opportunity to share our observations of different clients and discuss strategies for addressing the need of each individual.”*

### Comparison of outcomes based on learners’ characteristics

Tables 3 and 4 compares the study outcomes at baseline and the within-group changes in these outcomes on the basis of learners’ characteristics. At baseline, there were significant differences in the DKAS scores amongst different age group (*p* = .002), with the highest in those aged between 55 and 64, and followed by those aged between 25 and 44. The differences in the SNCW scores and the SCIDS scores across age groups were also statistically significant (*P*s ≤ .001), with the middle aged group (aged between 35 and 64) had relatively higher scores. Significant group differences were found in all outcomes amongst different occupations (*P*s ≤ 0.001). The scores of the clerical staff and supporting staff were generally lower than the other groups, suggesting poorer knowledge and more negative attitudes toward dementia. Staff members who were not involved in direct care for people with dementia and had not received any training before this project obtained significantly lower scores in all outcomes (*P*s ranged from ≤ .001 to 0.012).

**Table 3:** Comparison of outcome variables based on learners’ characteristics at baseline (*N* = 1069)

	DKAS – Total score			DAS – Total score			SNCW – Total score			SCIDS – Total score		
	Mean (*SD*)	*F/t*	*p*	Mean (*SD*)	*F/t*	*p*	Mean (*SD*)	*F/t*	*p*	Mean (*SD*)	*F/t*	*p*
Gender												
Male	28.4 (8.6)	.847	.397^†^	100.6 (12.9)	−1.84	.066^†^	117.5 (14.4)	−1.98	.051^†^	42.3 (6.8)	−1.90	.058^†^
Female	27.7 (8.3)			102.6 (11.7)			120.0 (13.6)			43.7 (8.3)		
Age groups		3.89	.002^‡^		0.59	.707^‡^		9.94	< .001^‡^		6.41	< .001^‡^
≤ 24	26.0 (8.4)			101.5 (8.9)			116.7 (13.0)			42.4 (6.9)		
25–34	28.6 (8.0)			101.6 (13.3)			115.4 (13.0)			41.5 (7.1)		
35–44	28.8 (9.2)			102.9 (11.8)			120.6 (13.4)			43.6 (7.4)		
45–54	26.6 (8.1)			102.5 (11.4)			122.4 (14.0)			44.9 (8.3)		
55–64	29.1 (8.0)			103.3 (11.6)			121.7 (13.3)			44.5 (8.1)		
≥ 65	27.6 (8.2)			101.8 (15.1)			116.4 (18.2)			44.0 (14.7)		
Types of staff		21.36	< .001^‡^		6.16	< .001^‡^		10.48	< .001^‡^		29.14	< .001^‡^
Care assistants	26.9 (7.8)			103.0 (11.7)			122.1 (12.8)			45.0 (7.6)		
Supporting staff	20.0 (7.7)			98.9 (12.2)			120.4 (17.1)			41.4 (8.0)		
Clerical staff	24.4 (9.1)			96.2 (11.6)			112.7 (12.4)			35.7 (9.5)		
Professional staff	31.2 (8.0)			103.0 (11.9)			117.1 (13.9)			42.7 (7.1)		
Managerial staff	28.5 (10.0)			102.6 (12.8)			117.7 (14.5)			43.7 (7.7)		
Provision of direct services to people with dementia in daily job duties		2.53	.012		4.91	< .001^†^		4.85	< .001^†^		7.12	< .001^†^
Yes	28.2 (8.3)			103.1 (11.6)			120.5 (13.5)			44.3 (7.6)		
No	26.3 (8.5)			97.9 (12.7)			114.5 (14.3)			39.3 (8.6)		
Ever received dementia care training before this study		41.69	< .001^‡^		47.53	< .001^‡^		18.32	< .001^‡^		30.61	< .001^‡^
Yes, more than once	30.8 (8.2)			106.2 (11.8)			122.3 (13.0)			45.4 (7.8)		
Yes, once only	28.1 (7.7)			103.5 (10.4)			120.8 (13.7)			44.8 (7.7)		
No	25.1 (8.2)			97.8 (11.9)			116.2 (14.0)			41.0 (7.9)		

Footnote: *N* total number, *SD* standard deviation, *DKAS* The Dementia Knowledge Assessment Scale, *DAS* Dementia Attitudes Scale, *SCIDS* The Sense of Competence in Dementia Care, *SNCW* Satisfaction with Nursing Care and Work Assessment Scale; ^†^*p value* of the *t*-statistic by independent sample-t test; ^‡^*p value* of the *F*-statistic by ANOVA test

**Table 4:** Comparison of changes in outcomes based on learners’ characteristics (*N* = 1069)

	Change of DKAS – Total score			Change of DAS – Total score			Change of SNCW – Total score			Change of SCIDS – Total score		
	Mean (*SD*)	*F/t*	*p*	Mean (*SD*)	*F/t*	*p*	Mean (*SD*)	*F/t*	*p*	Mean (*SD*)	*F/t*	*p*
Gender		0.13	.900^†^		1.95	.140 ^†^		−0.79	.429 ^†^		−0.78	.490^†^
Male	5.0 (7.3)			11.1 (12.2)			4.8 (12.0)			4.4 (6.7)		
Female	4.9 (7.9)			9.6 (11.2)			5.6 (12.3)			4.8 (6.9)		
Age groups		2.29	.044^‡^		3.73	.019 ^‡^		1.41	.274^‡^		4.27	< .001^‡^
≤ 24	4.5 (7.3)			8.7 (10.2)			5.0 (10.3)			4.3 (6.7)		
25–34	5.4 (7.6)			11.9 (12.0)			6.8 (12.1)			6.4 (6.8)		
35–44	4.9 (7.9)			9.8 (10.4)			4.3 (9.5)			5.0 (7.3)		
45–54	5.3 (8.2)			8.9 (10.7)			5.1 (12.6)			4.0 (7.0)		
55–64	3.4 (7.5)			8.4 (11.3)			6.5 (12.3)			3.7 (6.3)		
≥ 65	0.9 (7.0)			6.9 (15.8)			4.1 (10.2)			3.0 (3.3)		
Types of staff		3.22	.007^‡^		4.20	< .001^‡^		4.53	.011^‡^		18.15	< .001^‡^
Care assistants	4.9 (8.0)			8.9 (11.2)			5.2 (12.0)			4.0 (6.9)		
Supporting staff	6.3 (7.2)			5.1 (10.4)			2.5 (7.3)			3.6 (7.9)		
Clerical staff	6.3 (8.5)			12.0 (11.5)			2.2 (9.8)			7.3 (6.4)		
Professional staff	4.7 (7.1)			11.7 (11.0)			7.2 (11.7)			6.1 (6.7)		
Managerial staff	1.0 (7.3)			5.7 (8.4)			0.8 (9.0)			2.5 (7.2)		
Provision of direct services to people with dementia in daily job duties		−3.75	< .001^†^		−4.03	< .001 ^†^		−1.16	.248 ^†^		−3.60	< .001^†^
Yes	4.4 (7.7)			9.2 (11.0)			5.3 (12.2)			4.4 (6.9)		
No	7.1 (8.1)			13.6 (12.4)			6.6 (11.7)			6.6 (6.6)		
Ever received dementia care training before this study		16.64	< .001^‡^		12.31	< .001^‡^		1.81	.319^‡^		8.06	< .001^‡^
Yes, more than once	3.8 (7.4)			8.4 (9.8)		.	5.8 (11.4)			3.9 (6.7)		
Yes, once only	3.9 (7.7)			8.7 (10.3)			4.8 (11.8)			4.1 (6.8)		
No	6.8 (8.1)			12.5 (12.8)			6.2 (11.6)			6.1 (7.2)		

Footnote: *N* total number, *SD* standard deviation, *DKAS* The Dementia Knowledge Assessment Scale, *DAS* Dementia Attitudes Scale, *SCIDS* The Sense of Competence in Dementia Care, *SNCW* Satisfaction with Nursing Care and Work Assessment Scale; ^†^*p value* of the *t*-statistic by independent sample-t test; ^‡^*p value* of the *F*-statistic by ANOVA test

The changes in the outcomes were not associated with the gender of the learners. Except for the SNCW score, significant differences were noted amongst different age groups in the changes in the DKAS score (*p* = .044), the DAS score (*p* = .019) and the SCID score (*p* ≤ .001). Smaller changes were observed among those aged 55 years or above in these three study outcomes when compared with their younger counterparts. Significant differences were noted amongst staff members with different occupations in the DKAS score *(p* = .007), the DAS score *(p* ≤ .001), the SCID score (*p* ≤ .001) and SNCW total scores (*p* = .011). The managerial staff demonstrated the least improvement in all outcomes, whereas changes were high among the clerical staff in the DAS score and the SCIDS score, indicating improvement in attitude and sense of competence. Improvement in the DKAS scores were higher in clerical staff and supporting staff members in comparison with other groups. Although the DKAS score did not apparently increase amongst the professional staff, improvement in the DAS score, SNCW score and SCIDS score were generally high. Except for the SNCW score, the learners who have not been involved in dementia care and never received dementia training demonstrated significantly greater improvements across all outcomes (*P*s ≤ .001) than those who have received certain kinds of training or involved in direct care for people with dementia.

## Discussion

This study is the largest reported in the field of building dementia care workforce across different community care settings. Significant improvements were observed in all outcomes concerning staff knowledge, attitudes and sense of competence in dementia care, and job satisfaction at the 12-month follow-up assessment. The findings of the current study provide a relatively detailed examination of the training effects on different outcomes and staff members than the previous study [13]. The analysis on the changes in outcomes further showed that the effects of the training program significantly varied across different groups of learners in terms of age, occupations, work, and training experience. The findings contribute new knowledge to the field in terms of different training needs amongst staff members of various roles or qualifications.

The remarkable improvements of clerical and supporting staff on various outcomes, including knowledge, attitude, and sense of competence, suggested the training needs of non-care related staff to enhance their awareness toward dementia. The results are consistent with those of Adler et al.’s (2015) that supporting staff had lower levels of dementia knowledge and skills than the health care professionals [3]. The results also showed the knowledge gaps in staff members who had not been involved in dementia care or received prior relevant training. The training needs about dementia care of staff members who are not involved in direct care services but working within care settings are ignored. Previous studies on dementia care training mainly focused on health professionals, direct care workers, and healthcare students [2, 5, 6, 8, 14, 24]. Given that people with dementia interact with different staff members in the care environment, their reactions and responses would also be influential to care recipients’ wellbeing and thus care quality. Therefore, an inclusive approach to enhance the training impacts on care culture is warranted [10, 25]. The success of the present training program may be partly because it follows several key recommendations of teamwork education drawn from a meta-synthesis, including participation of all members, understanding on how the team function, opportunities for practice as well as reflection and debriefing [26]. Given that this project offered on-site training delivered by staff members trained as facilitators, the training focus could be tailored according to the context of their workplace and the learners could also directly apply the newly learned knowledge and skills in their real-world practice.

The substantial improvements in attitudes, sense of competence and job satisfaction of professional staff and care assistants were also noteworthy, given that their scores were amongst the top of all learners at baseline. These findings are in line with the literature that training is effective in increasing staff sense of competence in dementia care by improving their understanding about the challenging behaviors of people with dementia [7]. However, the findings disprove the conclusion of a systematic review that staff training has limited impact on care providers’ attitudes or job satisfaction [7]. The promising results of this study may be partly explicable in terms of the program design with continual sessions. Surr’s (2017) suggested that the consolidated time for training over a longer term is needed for attitudinal change [8]. The notable positive change in attitude can also be attributed to reflective learning, which invoked a careful consideration and questioning of one’s own beliefs, attitudes and values with respect to the existing care practices, thereby promoting self-awareness [15].

Third, the least improvements were observed generally in the managerial staff and those who were over 55 years old. The scores of the managerial staff were comparable with the professional staff at baseline. This can largely be explained by the fact that they generally possessed health and social care professional qualification. The subtle within-group changes could suggest that their training needs might be different from those working at frontline. Such observation was not noted in previous studies because the staff members of the managerial grades were not analysed separately [8, 25]. Given that organisational and managerial support is often regarded an a contributing factor to the implementation of dementia training [7, 25, 27], the findings seem to suggest the need for devising a specific program to address the training needs of this staff group. On the other hand, the insignificant changes of the older counterparts would need further investigations. The results may possibly relate to their job nature rather than age itself because their baseline scores were at both ends.

We acknowledged several study limitations. First, self-report measures and reflective essays were used to detect and explain changes. Second, the sustained effects of the training programs could not be ascertained yet because the follow-up assessment was conducted after 12 months, which is approximately 6 months after the training. Moreover, there was no control group for comparison, and the outcomes for the people with dementia and care quality were not collected. To enhance the credibility of the results, validated instruments were used in this study. In addition, the reflective essays were not graded, and the database was only accessible to the researchers who were external to the participants’ workplaces to prevent biased responses. We are collecting the third wave of data as the 24-month follow-up to examine the program effects using latent variable evaluation approach. Given the difficulty of adopting conventional research designs, future research can focus on the implementation sciences or comparison of the different modes of training on practice change and thus the influence on the care service for people with dementia and the satisfaction of their family members with regard to the service.

## Conclusions

Building the capacity of health and social care workforce for dementia care is at the top of the policy agenda worldwide [1]. This project provided a foundation for enhancing knowledge, attitudes, sense of competence in dementia care, and job satisfaction through an in-service dementia training program amongst different staff groups across community care and care home settings.

The promising results can be attributed to several crucial elements in the design of the training program, which aimed to create a supportive environment for in-service learning. First, the train-the-trainer model increased the extensiveness and cost-effectiveness of the training to a wider society and cultivated an atmosphere for workplace learning. The trained facilitators were the key to supporting on-going situated learning. Second, an inclusive approach was employed so that all staff members, including non-care related staff who were often neglected in relevant training, were involved to support and enhance the sustainability of practice and cultural changes, notwithstanding high staff turnover. The program also highlighted the importance of reflective learning. Rather than didactic teaching, regular face-to-face training sessions were filled with interactive activities and group discussions that encouraged learners to reflect upon the current dementia care practices in their workplace and appreciate how their newly learnt knowledge can inform practices. The reflective learning approach enabled the learners to actively develop practical knowledge in bridging the gap between theoretical knowledge and the reality in care settings. Instead of teaching one-size-fits-all management strategies, the learners had to exercise their own judgment in deciding which strategies would well address individual concerns on the basis of the principles of person-centered care.

## Data Availability

The datasets used and/or analyzed during the current study are available from the corresponding author on reasonable request.

## Abbreviations

DKAS: Dementia knowledge assessment scale
DAS: Dementia attitudes scale
SNCW: Satisfaction with nursing care and work assessment scale
SCIDS: Sense of competence in dementia care staff
MCAR: missing completely at random
ANOVA: one-way analysis of variance
